# 5-(1,3-Dithiolo[4,5-*d*][1,3]dithiol-2-yl­idene)-1,3-dithiolo[4,5-*c*][1,2,5]thia­diazole: an unsymmetrical tetra­thia­fulvalene with fused 1,2,5-thia­diazole and 1,3-dithiole rings

**DOI:** 10.1107/S1600536809014184

**Published:** 2009-04-22

**Authors:** Masaaki Tomura, Yoshiro Yamashita

**Affiliations:** a Institute for Molecular Science, Myodaiji, Okazaki 444-8585, Japan; b Department of Electronic Chemistry, Interdisciplinary Graduate School of Science and Engineering, Tokyo Institute of Technology, Nagatsuta, Midori-ku, Yokohama 226-8502, Japan

## Abstract

The title unsymmetrical tetra­thia­fulvalene (TTF), C_7_H_2_N_2_S_7_, contains fused 1,2,5-thia­diazole and 1,3-dithiole rings and is a component mol­ecule for conducting organic solids. The TTF mol­ecule is disordered crystallographically over two orientations related by an inversion center, where each site is half-occupied. The mol­ecule is almost planar with an r.m.s. deviation of 0.096 Å. In the crystal structure, mol­ecules are linked by short inter­molecular S⋯S inter­actions [3.47 (2), 3.507 (8) and 3.517 (13) Å].

## Related literature

For general background, see: Williams *et al.* (1992 ▶); Ishiguro *et al.* (1998 ▶); Yamashita & Tomura (1998 ▶). For the synthesis of the title compound, see: Tomura & Yamashita (1997 ▶). For unsymmetrical TTF derivatives with a fused 1,2,5-thia­diazole ring, see: Tomura *et al.* (1993 ▶); Underhill *et al.* (1993 ▶); Naito *et al.* (1996 ▶); Tomura & Yamashita (2003 ▶); Tomura & Yamashita (2004 ▶). For values of van der Waals radii, see: Bondi (1964 ▶).
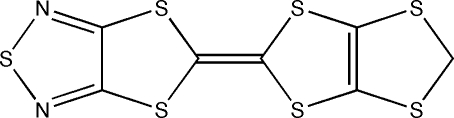


## Experimental

### 

#### Crystal data

C_7_H_2_N_2_S_7_
*M* *_r_* = 338.60
Monoclinic, 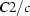

*a* = 27.42 (3) Å
*b* = 4.051 (3) Å
*c* = 11.047 (10) Å
β = 113.020 (15)°
*V* = 1129.4 (18) Å^3^
*Z* = 4
Mo *K*α radiation
μ = 1.36 mm^−1^
*T* = 291 K
0.10 × 0.05 × 0.01 mm

#### Data collection

Rigaku/MSC Mercury CCD diffractometer
Absorption correction: none
4788 measured reflections
1639 independent reflections
737 reflections with *I* > 2σ(*I*)
*R*_int_ = 0.176

#### Refinement

*R*[*F* ^2^ > 2σ(*F* ^2^)] = 0.052
*wR*(*F* ^2^) = 0.131
*S* = 0.84
1639 reflections
146 parameters
37 restraints
H-atom parameters constrained
Δρ_max_ = 0.42 e Å^−3^
Δρ_min_ = −0.41 e Å^−3^

### 

Data collection: *CrystalClear* (Rigaku/MSC, 2006 ▶); cell refinement: *CrystalClear*; data reduction: *TEXSAN* (Rigaku, 2004 ▶); program(s) used to solve structure: *SHELXS97* (Sheldrick, 2008 ▶); program(s) used to refine structure: *SHELXL97* (Sheldrick, 2008 ▶); molecular graphics: *PLATON* (Spek, 2009 ▶); software used to prepare material for publication: *SHELXL97*.

## Supplementary Material

Crystal structure: contains datablocks global, I. DOI: 10.1107/S1600536809014184/hb2951sup1.cif

Structure factors: contains datablocks I. DOI: 10.1107/S1600536809014184/hb2951Isup2.hkl

Additional supplementary materials:  crystallographic information; 3D view; checkCIF report

## Figures and Tables

**Fig. 1.**
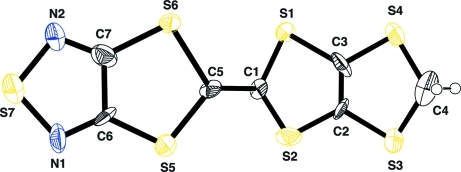
The molecular structure of (I), with atom labels and 50% probability displacement ellipsoids for non-H atoms and H atoms are shown as small spheres of arbitrary radii. One component of the disordered molecule is shown.

**Fig. 2.**
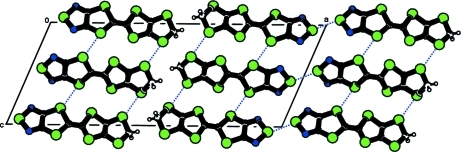
The packing diagram of (I), viewed along the b axis. Dashed lines indicate intermolecular S···S interactions. One component of the disordered molecule is shown.

## supplementary crystallographic information

### Comment

Tetrathiafulvalene (TTF) derivatives with a fused 1,2,5-thiadiazole ring have received much attention as component molecules for conducting organic solids (Tomura *et al.*, 1993; Underhill *et al.*, 1993; Naito *et al.*, 1996; Tomura & Yamashita, 2003; Tomura & Yamashita, 2004). Intermolecular interactions caused by S···N and S···S heteroatom contacts may increase the dimensionality in solid states and suppress metal-insulator transitions (Williams *et al.*, 1992; Ishiguro *et al.*, 1998). In addition, such interactions may lead to the formation of unique molecular networks which have special functions such as inclusion properties (Yamashita & Tomura, 1998). We report here the molecular and crystal structure of an unsymmetrical TTF derivative (I), which contains fused 1,2,5-thiadiazole and 1,3-dithiole rings (Fig. 1).

The center of the unsymmetrical TTF molecule (I) is located on an inversion center. Thus, the molecule is disordered crystallographically over two orientations related by the inversion center. Each site is half-occupied and the total site occupation factor (s.o.f.) equals 1.0. This type of disorder was not observed in the crystal of the unsymmetrical tetrathiafulvalene with fused 1,2,5-thiadiazole and 2,3-dihydro-1,4-dioxine rings (Tomura & Yamashita, 2003). Geometric resemblance between 5-membered 1,2,5-thiadiazole and 1,3-dithiole rings causes this disorder in the crystal of (I). Superlattice reflection was not observed on CCD images. This fact also suggests crystallographic disorder in the crystal.  The molecular framework is almost planar with an r.m.s. deviation of 0.096 Å from the least-squares plane. The [1,3]dithiolo[4,5-*c*][1,2,5]thiadiazole framework (S5/S6/S7/N1/N2/C5/C6/C7) is also planar with an r.m.s. deviation of 0.045 Å, while an r.m.s. deviation for the [1,3]dithiolo[4,5-*d*][1,3]dithiole plane (S1/S2/S3/S4/C1/C2/C3/C4) is large (0.104 Å). The deviations of S3, S4 and C4 atoms from the plane are -0.11 (1), -0.18 (1) and 0.18 (2) Å, respectively. The angle between the two plane is 3.0 (9)°. In the crystal structure, the molecules are linked *via* short intermolecular S···S interactions [3.517 (13) for S3—S4(*x*, -*y*, *z* - 1/2), 3.47 (2) for S5—S6(*x*, -*y*, *z* - 1/2) and 3.507 (8) Å for S7—S7(-*x*, -*y*, -*z*)] (Fig. 2). The S···S interactions are 2.3–3.6% shorter than the sum of the corresponding van der Waals radii (Bondi, 1964). No short intermolecular S···N interaction was observed.

### Experimental

The title compound was synthesized according to the literature method (Tomura & Yamashita, 1997). Greenish-brown plates of (I) were grown from a toluene solution.

### Refinement

The molecule (I) was located on an inversion center and was disordered crystallographically over two orientations related by the inversion center. Thus, the occupancy of all atoms was fixed to 0.5. All the H atoms were placed in geometrically calculated positions and refined using a riding model, with C—H = 0.97 Å and *U*_iso_(H) = 1.2*U*_eq_(C).   The refinement was slightly unstable, with some oscillating paramter shifts.

### Crystal data

**Table:**

C_7_H_2_N_2_S_7_	*F*(000) = 680
*M**_r_* = 338.60	*D*_x_ = 1.991 Mg m^−^^3^
Monoclinic, *C*2/*c*	Mo *K*α radiation, λ = 0.71070 Å
Hall symbol: -C 2yc	Cell parameters from 1272 reflections
*a* = 27.42 (3) Å	θ = 2.0–30.3°
*b* = 4.051 (3) Å	µ = 1.36 mm^−^^1^
*c* = 11.047 (10) Å	*T* = 291 K
β = 113.020 (15)°	Platelet, green–brown
*V* = 1129.4 (18) Å^3^	0.10 × 0.05 × 0.01 mm
*Z* = 4	

### Data collection

**Table:**

Rigaku/MSC Mercury CCD diffractometer	737 reflections with *I* > 2σ(*I*)
Radiation source: Rotating Anode	*R*_int_ = 0.176
Confocal	θ_max_ = 31.0°, θ_min_ = 3.2°
Detector resolution: 14.63 pixels mm^-1^	*h* = −39→35
φ and ω scans	*k* = −5→5
4788 measured reflections	*l* = −15→15
1639 independent reflections	

### Refinement

**Table:**

Refinement on *F*^2^	Secondary atom site location: difference Fourier map
Least-squares matrix: full	Hydrogen site location: inferred from neighbouring sites
*R*[*F*^2^ > 2σ(*F*^2^)] = 0.052	H-atom parameters constrained
*wR*(*F*^2^) = 0.131	*w* = 1/[σ^2^(*F*_o_^2^) + (0.0051*P*)^2^] where *P* = (*F*_o_^2^ + 2*F*_c_^2^)/3
*S* = 0.84	(Δ/σ)_max_ = 0.135
1639 reflections	Δρ_max_ = 0.42 e Å^−^^3^
146 parameters	Δρ_min_ = −0.40 e Å^−^^3^
37 restraints	Extinction correction: *SHELXL97* (Sheldrick, 2008), Fc^*^=kFc[1+0.001xFc^2^λ^3^/sin(2θ)]^-1/4^
Primary atom site location: structure-invariant direct methods	Extinction coefficient: 0.0064 (8)

### Special details

**Table:**

Geometry. All e.s.d.'s (except the e.s.d. in the dihedral angle between two l.s. planes) are estimated using the full covariance matrix. The cell e.s.d.'s are taken into account individually in the estimation of e.s.d.'s in distances, angles and torsion angles; correlations between e.s.d.'s in cell parameters are only used when they are defined by crystal symmetry. An approximate (isotropic) treatment of cell e.s.d.'s is used for estimating e.s.d.'s involving l.s. planes.
Refinement. Refinement of *F*^2^ against ALL reflections. The weighted *R*-factor *wR* and goodness of fit *S* are based on *F*^2^, conventional *R*-factors *R* are based on *F*, with *F* set to zero for negative *F*^2^. The threshold expression of *F*^2^ > σ(*F*^2^) is used only for calculating *R*-factors(gt) *etc*. and is not relevant to the choice of reflections for refinement. *R*-factors based on *F*^2^ are statistically about twice as large as those based on *F*, and *R*- factors based on ALL data will be even larger.

### Fractional atomic coordinates and isotropic or equivalent isotropic displacement parameters (Å^2^)

**Table:**

	*x*	*y*	*z*	*U*_iso_*/*U*_eq_	Occ. (<1)
S1	0.3251 (4)	0.093 (4)	0.1411 (15)	0.045 (2)	0.50
S2	0.2922 (4)	0.410 (3)	−0.1189 (10)	0.0329 (12)	0.50
S3	0.4100 (3)	0.388 (3)	−0.0802 (9)	0.0541 (13)	0.50
S4	0.4378 (2)	0.0369 (16)	0.1767 (6)	0.0487 (12)	0.50
C1	0.2792 (6)	0.222 (4)	0.0102 (19)	0.026 (2)	0.50
C2	0.3615 (8)	0.325 (7)	−0.018 (2)	0.045 (5)	0.50
C3	0.3735 (8)	0.185 (13)	0.091 (4)	0.037 (5)	0.50
C4	0.4602 (7)	0.284 (6)	0.0755 (16)	0.114 (12)	0.50
H4A	0.4752	0.4862	0.1222	0.137*	0.50
H4B	0.4883	0.1668	0.0609	0.137*	0.50
S5	0.1724 (4)	0.394 (4)	−0.1612 (15)	0.0391 (17)	0.50
S6	0.2003 (4)	0.029 (3)	0.0999 (10)	0.0320 (11)	0.50
S7	0.04227 (19)	0.2329 (14)	−0.0516 (6)	0.0621 (12)	0.50
C5	0.2283 (7)	0.206 (4)	−0.0082 (19)	0.028 (3)	0.50
C6	0.1193 (8)	0.320 (12)	−0.102 (4)	0.042 (5)	0.50
C7	0.1363 (6)	0.153 (7)	0.031 (2)	0.039 (5)	0.50
N1	0.0679 (7)	0.362 (5)	−0.1582 (19)	0.052 (5)	0.50
N2	0.0974 (7)	0.090 (9)	0.067 (2)	0.054 (6)	0.50

### Atomic displacement parameters (Å^2^)

**Table:**

	*U*^11^	*U*^22^	*U*^33^	*U*^12^	*U*^13^	*U*^23^
S1	0.054 (3)	0.058 (3)	0.021 (4)	0.000 (2)	0.013 (2)	0.002 (3)
S2	0.025 (3)	0.045 (4)	0.021 (3)	−0.0144 (17)	0.0004 (19)	−0.0080 (17)
S3	0.038 (2)	0.090 (3)	0.0441 (17)	−0.002 (2)	0.0270 (17)	0.005 (2)
S4	0.0308 (14)	0.068 (3)	0.0469 (17)	0.0016 (18)	0.0152 (12)	0.004 (2)
C1	0.027 (4)	0.023 (6)	0.030 (4)	0.015 (4)	0.014 (3)	−0.006 (4)
C2	0.045 (9)	0.064 (12)	0.041 (8)	−0.021 (7)	0.034 (7)	−0.012 (7)
C3	0.012 (4)	0.061 (13)	0.030 (7)	0.013 (5)	−0.002 (4)	0.005 (8)
C4	0.038 (10)	0.18 (3)	0.113 (18)	0.006 (13)	0.012 (9)	0.035 (17)
S5	0.0292 (19)	0.070 (4)	0.017 (3)	0.0054 (19)	0.0081 (15)	0.005 (2)
S6	0.025 (3)	0.044 (4)	0.020 (3)	−0.0109 (18)	0.002 (2)	−0.0072 (17)
S7	0.040 (2)	0.084 (3)	0.0738 (19)	0.008 (2)	0.0353 (16)	0.020 (2)
C5	0.031 (5)	0.026 (7)	0.027 (4)	0.020 (4)	0.012 (4)	0.008 (4)
C6	0.047 (11)	0.054 (15)	0.028 (11)	−0.008 (11)	0.020 (10)	−0.002 (8)
C7	0.024 (7)	0.055 (10)	0.027 (6)	0.017 (7)	−0.002 (5)	0.017 (6)
N1	0.034 (6)	0.070 (12)	0.060 (9)	0.007 (6)	0.029 (6)	0.013 (6)
N2	0.022 (5)	0.105 (11)	0.042 (8)	0.001 (6)	0.019 (4)	−0.002 (7)

### Geometric parameters (Å, °)

**Table:**

S1—C3	1.67 (4)	C4—H4B	0.9700
S1—C1	1.59 (2)	S5—C5	1.94 (2)
S2—C1	1.77 (3)	S5—C6	1.84 (3)
S2—C2	1.82 (2)	S6—C7	1.694 (18)
S3—C2	1.739 (12)	S6—C5	1.80 (3)
S3—C4	1.784 (13)	S7—N2	1.669 (15)
S4—C3	1.749 (13)	S7—N1	1.674 (13)
S4—C4	1.779 (13)	C6—N1	1.311 (14)
C1—C5	1.331 (8)	C6—C7	1.52 (4)
C2—C3	1.25 (4)	C7—N2	1.300 (14)
C4—H4A	0.9700		
			
S3···S4^i^	3.517 (13)	S7···S7^ii^	3.507 (8)
S5···S6^i^	3.47 (2)		
			
C3—S1—C1	94.3 (15)	S4—C4—H4B	108.7
C1—S2—C2	85.1 (9)	H4A—C4—H4B	107.6
C2—S3—C4	90.2 (10)	C5—S5—C6	95.4 (12)
C3—S4—C4	89.5 (16)	C7—S6—C5	102.7 (10)
C5—C1—S1	122.1 (10)	N2—S7—N1	99.2 (9)
C5—C1—S2	115.3 (7)	C1—C5—S6	127.7 (7)
S1—C1—S2	122.5 (12)	C1—C5—S5	122.6 (8)
C3—C2—S3	120 (2)	S6—C5—S5	109.7 (10)
C3—C2—S2	119.6 (16)	N1—C6—C7	112 (3)
S3—C2—S2	120.2 (13)	N1—C6—S5	132 (3)
C2—C3—S1	118.4 (13)	C7—C6—S5	115.5 (11)
C2—C3—S4	120 (3)	N2—C7—C6	114.0 (17)
S1—C3—S4	121 (2)	N2—C7—S6	129.3 (15)
S3—C4—S4	114.4 (11)	C6—C7—S6	116.1 (13)
S3—C4—H4A	108.6	C6—N1—S7	107 (2)
S4—C4—H4A	108.6	C7—N2—S7	107.0 (13)
S3—C4—H4B	108.7		
			
C3—S1—C1—C5	179 (2)	S1—C1—C5—S5	175.1 (18)
C3—S1—C1—S2	−3(3)	S2—C1—C5—S5	−3.0 (11)
C2—S2—C1—C5	−179.7 (11)	C7—S6—C5—C1	170.1 (13)
C2—S2—C1—S1	2.3 (17)	C7—S6—C5—S5	−7.9 (15)
C4—S3—C2—C3	−12 (4)	C6—S5—C5—C1	−172.9 (18)
C4—S3—C2—S2	175.9 (19)	C6—S5—C5—S6	5(2)
C1—S2—C2—C3	0(4)	C5—S5—C6—N1	−174 (4)
C1—S2—C2—S3	171.8 (19)	C5—S5—C6—C7	0(3)
S3—C2—C3—S1	−174 (3)	N1—C6—C7—N2	−2(5)
S2—C2—C3—S1	−2(6)	S5—C6—C7—N2	−177 (3)
S3—C2—C3—S4	−3(6)	N1—C6—C7—S6	170 (3)
S2—C2—C3—S4	169 (2)	S5—C6—C7—S6	−5(4)
C1—S1—C3—C2	3(5)	C5—S6—C7—N2	179 (3)
C1—S1—C3—S4	−168 (3)	C5—S6—C7—C6	8(3)
C4—S4—C3—C2	16 (5)	C7—C6—N1—S7	4(4)
C4—S4—C3—S1	−173 (4)	S5—C6—N1—S7	178 (4)
C2—S3—C4—S4	22.8 (18)	N2—S7—N1—C6	−4(3)
C3—S4—C4—S3	−24 (2)	C6—C7—N2—S7	−1(4)
S1—C1—C5—S6	−2.8 (14)	S6—C7—N2—S7	−172 (2)
S2—C1—C5—S6	179.2 (14)	N1—S7—N2—C7	3(3)

Symmetry codes: (i) *x*, −*y*, *z*−1/2; (ii) −*x*, −*y*, −*z*.
